# Knowledge of healthcare professionals about poliomyelitis and postpoliomyelitis: a cross-sectional study

**DOI:** 10.1590/1516-3180.2020.0617.16032021

**Published:** 2021-08-09

**Authors:** Claudio Andre Barbosa de Lira, Douglas Assis Teles Santos, Ricardo Borges Viana, Juliana Moreira Guimarães, Jéssica Nathalia Soares Oliveira, Bolivar Saldanha Sousa, Marcos Gonçalves de Santana, Rodrigo Luiz Vancini, Marília Santos Andrade, Pantelis Nikolaidis, Thomas Rosemann, Beat Knechtle

**Affiliations:** I BSc, PhD. Associate Professor, Faculdade de Educação Física e Dança (FEFD), Universidade Federal de Goiás (UFG), Goiânia (GO), Brazil.; II BSc, MSc. Assistent Professor, Colegiado de Educação Física, Universidade do Estado da Bahia (UNEB), Teixeira de Freitas (BA), Brazil.; III BSc, PhD. Professor, Escola Superior de Educação Física e Fisioterapia do Estado de Goiás (ESEFFEGO), Universidade Estadual de Goiás (UEG), Goiânia (GO), Brazil.; IV BSc. Nurse, Unidade Acadêmica Especial de Ciências da Saúde, Universidade Federal de Jataí (UFJ), Jataí (GO), Brazil.; V BSc. Biomedic, Unidade Acadêmica Especial de Ciências da Saúde (UA-CISAU), Universidade Federal de Jataí (UFJ), Jataí (GO), Brazil.; VI MD, PhD. Physician, Hospital Israelita Albert Einstein, São Paulo-Brazil, São Paulo (SP), Brazil.; VII BSc, PhD. Associate Professor, Unidade Acadêmica Especial de Ciências da Saúde (UA-CISAU), Universidade Federal de Jataí (UFJ), Jataí (GO), Brazil.; VIII BSc, PhD. Adjunct Professor, Centro de Educação Física e Desportos, Universidade Federal do Espírito Santo (UFES), Vitória (ES), Brazil.; IX PhD. Physical Therapist and Associate Professor, Department of Physiology, Universidade Federal de São Paulo (USP), São Paulo (SP), Brazil.; X BSc, PhD. Professor, School of Health and Caring Sciences, University of West Attica, Athens, Greece.; XI MD, PhD. Physician and Professor, Institute of Primary Care, University of Zurich, Zurich, Switzerland.; XII MD, PhD. Physician and Scientific Assistant, Institute of Primary Care, University of Zurich, Zurich, Switzerland; and Researcher, Medbase St. Gallen Am Vadianplatz, St. Gallen, Switzerland

**Keywords:** Poliomyelitis, Physicians, Nervous system, Postpoliomyelitis syndrome, Knowledge, Medical education, Neuromuscular disease, Late effects of polio

## Abstract

**BACKGROUND:**

Postpoliomyelitis syndrome is a clinical condition that can affect poliomyelitis survivors.

**OBJECTIVE:**

Our aim was to evaluate knowledge of poliomyelitis and postpoliomyelitis syndrome among Brazilian healthcare professionals.

**DESIGN AND SETTING:**

Cross-sectional study conducted at a Brazilian public higher education institution located in the state of Goiás.

**METHODS:**

The participants (n = 578) were Brazilian physicians, physical therapists, nurses, nutritionists and psychologists. A self-administered questionnaire (30 questions) was designed to probe knowledge about poliomyelitis and postpoliomyelitis syndrome. From the questionnaire, we created a structured test to objectively evaluate the knowledge of these professionals. The test was composed of 20 questions and was scored over a range from 0 (totally ill-informed) to 20 (totally well-informed).

**RESULTS:**

In general, the physicians, physical therapists and nurses demonstrated better understanding of poliomyelitis and postpoliomyelitis syndrome. The healthcare professionals who had received previous information about poliomyelitis and postpoliomyelitis syndrome had significantly higher scores than those who had never received information (P < 0.001). On average, this difference was approximately 28.6%.

**CONCLUSIONS:**

The findings from the present study indicate that there is a critical need for improvement of knowledge about postpoliomyelitis syndrome among Brazilian healthcare professionals. The services provided by these professionals may therefore become compromised. Furthermore, public healthcare initiatives should be implemented to improve knowledge among healthcare professionals.

## INTRODUCTION

Poliomyelitis is an infectious viral disease that may attack people at any age. It affects the nervous system, resulting in paralysis and muscle spasms, and in some cases encephalitis.^1^^,^^2^ There are several regions in the world that are certified as free from poliomyelitis.^3^ However, there are some endemic countries, such as Afghanistan, Nigeria and Pakistan, and there are records of imported cases in some African countries.^3^ In Brazil, even though poliomyelitis has been eradicated, according to the Brazilian Health Ministry, 312 Brazilian municipalities have polio vaccine coverage of below 50%. This creates a state of alert and threatens the eradication of the disease. Therefore, there is a need to maintain permanent and effective actions of disease surveillance and adequate levels of immunological protection for the population.^4^

Postpoliomyelitis syndrome (PPS) is the term used to describe a collection of signs and symptoms that may be experienced by individuals afflicted by paralytic poliomyelitis after years of clinical and functional stability.^5^ The signs and symptoms that characterize PPS are new muscle weakness, muscle fatigue, muscle atrophy, muscle and joint pain, sleep disturbances, intolerance to cold, respiratory and swallowing difficulties and a recent increase in body mass.^6^^-^^8^ It is a slowly progressive disease, usually insidious, with subacute onset, sometimes resulting in significant restrictions of activities associated with everyday life.^7^,^9^^-^^16^

Although the pathophysiology of PPS is unclear, different mechanisms have been proposed. The most accepted mechanism postulates that degeneration or dysfunction of giant motor units, manifested by peripheral deterioration (axon and/or neuromuscular junction), probably as a result of metabolic requirements of giant motor units (muscle overuse), has a central role in the etiology of the disease.^9^^,^^17^ However, there are different hypotheses associated with the pathophysiology of the disease, which include: muscle disuse, the normal loss of motor units with age, predisposition to motor neuron degeneration due to glial vascular and lymphatic damage, reactivation of the virus or persistent infection, immunological factors related to poliomyelitis, ^9^^,^^15^^,^^18^^-^^20^ the effect of growth hormone and the combined effect of overuse, disuse, pain, body mass gain or other diseases.^9^^,^^18^^,^^20^

Regarding the diagnostic criteria for PPS, a clinical approach aimed at ruling out other neurological diseases, orthopedic conditions, psychiatric disorders or even consequences linked to the aging process is required, since these conditions could develop the same signs and symptoms as seen in PPS.^9^^,^^21^ Therefore, it is extremely important that patients should be managed by a multidisciplinary healthcare professional team that includes neurologists, rheumatologists, orthopedists, pulmonologists, physical education professionals, physical therapists, nutritionists, nurses and psychologists.^9^^,^^22^^,^^23^

Despite the significant decline in the incidence of paralytic poliomyelitis,^3^ PPS will remain a major health problem for many years. In western countries, where the last large epidemics date back to the 1940s and 1950s, many survivors of paralytic poliomyelitis are now aged between 70 and 80 years.^24^ In Brazil, the last major outbreak was in 1984.^24^ Therefore, the future outlook is for continued or even increased need for rehabilitation programs and management of people with PPS.^9^^,^^25^ In addition, according to the World Health Organization, it is estimated that there are about 18 million people alive who were affected by paralytic poliomyelitis.^3^

As it is reasonable to assume that success in the treatment of any disease depends on the knowledge that the healthcare professional has about the disease, assessment of knowledge about poliomyelitis and PPS among healthcare professionals is of great value. Previously, it was demonstrated that physical education professionals have misconceptions about poliomyelitis and PPS.^8^ Therefore, it is reasonable to assume that other healthcare professionals present similar misconceptions regarding poliomyelitis and PPS. Furthermore, other studies have shown that healthcare professionals can present misconceptions about epilepsy,^26^^,^^27^ acquired immune deficiency syndrome (AIDS),^28^ cancer,^29^ hypertension and diabetes.^30^

## OBJECTIVE

The aim of this study was to verify the knowledge of health professionals about poliomyelitis and PPS regarding the pathophysiology, etiology, symptomatology and forms of treatment of PPS.

## METHODS

### Participants

This was a cross-sectional study in which a total of 578 participants (454 women and 124 men) were recruited. This was done using different sources of advertisement (i.e. internet, local newspapers, magazines and billboards in universities, clinics, hospitals and gyms). The inclusion criterion for the study was that the participants should be professionals with at least an undergraduate degree in medicine, physical therapy, nursing, nutrition or psychology. Healthcare professionals with specialization in neurology and/or neuromuscular disorders were excluded from the study.

All the participants were informed of the aim of the study and experimental procedures, and written informed consent was obtained from each participant before any data were collected. All procedures involved in this study were approved by the Universidade Federal de Goiás (UFG) Ethics Committee (protocol number: 198/2009; approved on August 1, 2010) and followed the principles outlined in the Declaration of Helsinki.

### Questionnaire

To evaluate knowledge about poliomyelitis and PPS, we used the same questionnaire as described by de Lira et al.^8^ This was a self-administered questionnaire divided into three parts: (i) personal data; (ii) knowledge about poliomyelitis; and (iii) knowledge about PPS. The questionnaire consisted of 14 questions about poliomyelitis and 16 questions about PPS and the aspects of these conditions that were addressed related to pathophysiology, diagnosis, forms of treatment, prognosis and previous work experience, with simple scales for closed-type responses. This questionnaire had been prepared in accordance with previous recommendations.^31^^,^^32^ The questions are shown in Tables 2 and 3.

In addition, a knowledge assessment test was created. This aimed to objectively show the knowledge of the professionals interviewed. The test comprised 20 questions within the questionnaire, which were analyzed as a score for correct responses, ranging from 0 to 20 points. These questions are marked with an asterisk in Tables 2 and 3.

### Statistical analysis

Descriptive statistics were used to analyze the findings (mean, standard deviation and absolute and relative frequencies). The Gaussian distribution of the sample was tested by means of the Kolmogorov-Smirnov test. One-way analysis of variance (ANOVA) was used to compare age and professional experience among categories of healthcare professionals. The chi-square test was used to determine the association between healthcare professionals and knowledge about poliomyelitis and PPS. The Kruskal-Wallis test was used to compare scores from the questionnaire, obtained by the different healthcare professional categories, followed by Dunn post-hoc comparison. The Mann-Whitney test was used to compare scores obtained in the questionnaire between the exposure (‘yes’ and ‘no’) groups. For all statistical procedures, the significance level assumed was 5%.

## RESULTS

### Participants

A total of 578 participants (454 women and 124 men, comprising a convenience sample) were evaluated. Of these, 69 were physicians (19 women and 50 men), 151 were physical therapists (116 women and 35 men), 224 were nurses (203 women and 21 men), 78 were nutritionists (74 women and 4 men) and 56 were psychologists (42 women and 14 men). Out of the 578 participants approached, 305 (52.8%) had a specialization degree, 126 (21.8%) had a PhD, 86 (14.9%) had a master’s qualification, 59 (10.2%) only had an undergraduate degree and two (0.2%) did not report their highest graduation level. The other characteristics of the participants are presented in **Table 1**.

**Table 1:** Characteristics of participants (n = 578)

Variable (years)	Physicians n = 69	Physical therapists n = 151	Nurses n = 224	Nutritionists n = 78	Psychologists n = 56
Age	42.0 ± 13.4	32.1 ± 8.3^*^	33.7 ± 14.0^*^	30.3 ± 8.0^*^^†^	36.1 ± 11.5^*^
Professional experience	17.8 ± 12.6	8.2 ± 8.0^*^	7.9 ± 8.8^*^	7.3 ± 9.1^*^	11.2 ± 9.3^*^

Data are presented as mean ± standard deviation

^*^Statistically different from physicians (one-way analysis of variance (ANOVA) and Tukey post-test; P < 0.05)

^†^Statistically different from psychologists (one-way ANOVA and Tukey post-test; P < 0.05).

### Knowledge about poliomyelitis

The first part of the questionnaire was designed to assess knowledge about poliomyelitis. Out of the 578 participants approached, 576 (99.7%) had heard about poliomyelitis. Surprisingly, two nurses (0.35% of the participants) had never heard of poliomyelitis (**Table 2**). The chi-square test did not reveal any significant association between healthcare professional category and having heard about poliomyelitis (P = 0.530).

**Table 2:** Answers among healthcare professionals relating to poliomyelitis

Questions	Physicians n = 69 (%)	Physical therapists n = 151 (%)	Nurses n = 224 (%)	Nutritionists n = 78 (%)	Psychologists n = 56 (%)	P-value of χ^2^ test
Have you heard about poliomyelitis?						
Yes	69 (100)	151 (100)	222 (99.1)	78 (100)	56 (100)	0.004
No	0 (0.0)	0 (0.0)	2 (0.9)	0 (0.0)	0 (0.0)	
	a/b/c/d	a/e/f/g	b/e/h/i	c/f/h/j	d/g/i/j	
Have you had information about poliomyelitis (books, pamphlets and lectures)?						
Yes	64 (92.8)	137 (90.7)	198 (88.4)	34 (43.6)	28 (50)	< 0.001
No	5 (7.2)	14 (9.3)	26 (11.6)	44 (56.4)	28 (50)	
Did not know	0 (0.0)	0 (0.0)	0 (0.0)	0 (0.0)	0 (0.0)	
Did not answer	0 (0.0)	0 (0.0)	0 (0.0)	0 (0.0)	0 (0.0)	
	a/b	a/c	b/c	d	d	
Is poliomyelitis caused by a virus?^*^						
Yes	68 (98.6)	137 (90.7)	209 (93.3)	56 (71.8)	39 (69.6)	< 0.001
No	0 (0.0)	5 (3.3)	8 (3.6)	4 (5.1)	6 (10.7)	
Did not know	1 (1.4)	7 (4.6)	7 (3.10	18 (23.1)	11 (19.6)	
Did not answer	0 (0.0)	2 (1.3)	0 (0.0)	0 (0.0)	0 (0.0)	
	a/b/c	a/d/e	b/d/f	g	c/e/f/g	
Can poliomyelitis be spread through water and/or food contaminated with feces from a sick person?^*^						
Yes	54 (78.3)	60 (39.7)	122 (54.5)	17 (21.8)	14 (25)	< 0.001
No	10 (14.5)	57 (37.7)	72 (32.1)	42 (53.8)	14 (25)	
Did not know	5 (7.2)	34 (22.5)	30 (13.4)	19 (24.40	28 (50)	
Did not answer	0 (0.0)	0 (0.0)	0 (0.0)	0 (0.0)	0 (0.0)	
	a/b	a/c/d	b/c/e	d/e		
Can poliomyelitis cause gastrointestinal symptoms?^*^						
Yes	50 (72.5)	62 (41.1)	109 (48.7)	21 (26.9)	15 (26.8)	
No	8 (11.6)	25 (16.6)	61 (27.2)	25 (32.1)	8 (14.3)	
Did not know	11 (15.9)	63 (41.7)	54 (24.1)	32 (41)	33 (58.9)	
Did not answer	0 (0.0)	1 (0.7)	0 (0.0)	0 (0.0)	0 (0.0)	
	a/b	c/d	a/e	b/c/e	d/e	
Can poliomyelitis cause neuromuscular symptoms such as paralysis, paresis, muscle atrophy and weakness?^*^						
Yes	68 (98.6)	146 (96.7)	222 (99.1)	76 (97.4)	52 (92.9)	< 0.001
No	0 (0.0)	2 (1.3)	1 (0.4)	0 (0.0)	1 (1.8)	
Did not know	1 (1.4)	3 (2)	1 (0.4)	2 (2.6)	3 (5.4)	
Did not answer	0 (0.0)	0 (0.0)	0 (0.0)	0 (0.0)	0 (0.0)	
	a/b/c/d	a/e/f/g	b/e/h/i	c/f/h/j	d/g/i/j	
After the acute poliomyelitis stage, can patients recover functional capacity of affected structures fully or partially?^*^						
Yes	57 (82.6)	106 (70.2)	121 (54)	26 (33.3)	27 (48.2)	< 0.001
No	9 (13)	41 (27.2)	63 (28.1)	17 (21.8)	11 (19.6)	
Did not know	1 (1.4)	4 (2.6)	40 (17.9)	34 (43.6)	18 (32.1)	
Did not answer	2 (2.9)	0 (0.0)	0 (0.0)	1 (1.3)	0 (0.0)	
	a/b/c/d	a	b/e	c/f	d/e/f	
Has poliomyelitis been eradicated around the world?^*^						
Yes	9 (13)	36 (23.8)	53 (23.7)	17 (21.8)	11 (19.6)	< 0.001
No	55 (79.7)	96 (63.9)	148 (66.1)	50 (64.1)	36 (64.3)	
Did not know	5 (7.2)	19 (12.6)	23 (10.3)	11 (14.1)	9 (16.1)	
Did not answer	0 (0.0)	0 (0.0)	0 (0.0)	0 (0.0)	0 (0.0)	
	a/b	c/d/e	a/c/f/g	d/f/h	b/e/g/h	
Is there a vaccine available to prevent poliomyelitis?^*^						
Yes	69 (100)	148 (98)	220 (98.2)	74 (94.9)	53 (94.6)	0.002
No	0 (0.0)	1 (0.7)	2 (0.9)	0 (0.0)	0 (0.0)	
Did not know	0 (0.0)	2 (1.3)	2 (0.9)	4 (5.1)	3 (5.4)	
Did not answer	0 (0.0)	0 (0.0)	0 (0.0)	0 (0.0)	0 (0.0)	
	a/b/c	a/d/e/f	b/d/g/h	e/g/i	c/f/h/i	
Can poliomyelitis treatment involve admission to an intensive care unit, due to respiratory impairment?^*^						
Yes	65 (94.2)	119 (78.8)	165 (73.7)	26 (33.3)	17 (30.4)	< 0.001
No	2 (2.9)	6 (4)	20 (8.9)	2 (2.6)	4 (7.1)	
Did not know	2 (2.9)	26 (17.2)	39 (17.4)	50 (64.1)	35 (62.5)	
Did not answer	0 (0.0)	0 (0.0)	0 (0.0)	0 (0.0)	0 (0.0)	
	a	b	a/b	c	c	
Are you afraid to live with a person who had poliomyelitis?						
Yes	0 (0.0)	0 (0.0)	13 (5.8)	0 (0.0)	2 (3.6)	0.007
No	69 (100)	146 (96.7)	207 (92.4)	77 (98.7)	53 (94.6)	
Did not know	0 (0.0)	0 (0.0)	0 (0.0)	0 (0.0)	0 (0.0)	
Did not answer	0 (0.0)	5 (3.3)	4 (1.8)	1 (1.3)	1 (1.8)	
	a/b	c/d/e	c/f/g	a/d/f/h	b/e/g/h	
During your undergraduate course, did you have access to information on how to handle patients with poliomyelitis in your future profession?						
Yes	41 (59.4)	90 (59.6)	91 (40.6)	3 (3.8)	5 (8.9)	< 0.001
No	28 (40.6)	60 (39.7)	128 (57.1)	75 (96.2)	51 (91.1)	
Did not know	0 (0.0)	0 (0.0)	0 (0.0)	0 (0.0)	0 (0.0)	
Did not answer	0 (0.0)	1 (0.7)	5 (2.2)	0 (0.0)	0 (0.0)	
	a/b/c	a	b	c/d	d	
In your practice, have you ever provided a service for people with sequelae of poliomyelitis?						
Yes	48 (69.6)	78 (51.7)	82 (36.6)	10 (12.8)	12 (21.4)	< 0.001
No	21 (30.4)	69 (45.7)	137 (61.2)	68 (87.2)	43 (76.8)	
Did not know	0 (0.0)	0 (0.0)	0 (0.0)	0 (0.0)	0 (0.0)	
Did not answer	0 (0.0)	4 (2.6)	5 (2.2)	0 (0.0)	1 (1.8)	
	a	a	b	c	b/c	
Can people with sequelae of poliomyelitis perform any type of physical activity?^*^						
Yes	64 (92.8)	137 (90.7)	177 (79)	50 (64.1)	37 (66.1)	< 0.001
No	1 (1.4)	3 (2)	18 (8)	2 (2.6)	4 (7.1)	
Did not know	4 (5.8)	11 (7.3)	29 (12.9)	26 (33.3)	15 (26.8)	
Did not answer	0 (0.0)	0 (0.0)	0 (0.0)	0 (0.0)	0 (0.0)	
	a/b/c	a/d/e	b/d/f	g	c/e/f/g	

^*^ Question that composed the knowledge assessment test on paralytic poliomyelitis and PPS. Frequencies followed by the same letters, in the rows, did not differ.

Out of the 578 participants approached, 461 (79.8%) answered that they had received information about the disease through books, pamphlets and lectures. While approximately 90% of the physicians, physical therapists and nurses had had access to information on how to deal with poliomyelitis in their undergraduate courses, this proportion fell to approximately half among the nutritionists (43.6%) and psychologists (50.0%) (**Table 2**). The chi-square test revealed that there was a significant association between healthcare professional category and receiving information about poliomyelitis (P < 0.001).

**Table 3:** Answers among healthcare professionals relating to post-poliomyelitis syndrome (PPS)

Questions	Physicians n = 69 (%)	Physical therapists n = 151 (%)	Nurses n = 224 (%)	Nutritionists n = 78 (%)	Psychologists n = 56 (%)	P-value of χ^2^ test
Have you heard about PPS?						
Yes	42 (60.9)	102 (67.5)	78 (34.8)	10 (12.8)	11 (19.6)	< 0.001
No	26 (37.7)	29 (32.5)	145 (64.7)	68 (87.2)	45 (80.4)	
Did not know	0 (0.0)	0 (0.0)	0 (0.0)	0 (0.0)	0 (0.0)	
Did not answer	1 (1.4)	0 (0.0)	1 (0.4)	0 (0.0)	0 (0.0)	
	a	a		b	b	
Have you received information about PPS?						
Yes	37 (53.6)	82 (54.3)	59 (26.3)	5 (6.4)	8 (14.3)	< 0.001
No	32 (46.4)	69 (45.7)	159 (71)	72 (92.3)	47 (83.9)	
Did not know	0 (0.0)	0 (0.0)	0 (0.0)	0 (0.0)	0 (0.0)	
Did not answer	0 (0.0)	0 (0.0)	6 (2.7)	1 (1.3)	1 (1.8)	
	a	a	b	c	b/c	
Is PPS a disease that only affects patients who have had paralytic poliomyelitis?^*^						
Yes	23 (33.3)	64 (42.4)	65 (29)	15 (19.2)	9 (16.1)	< 0.001
No	19 (27.5)	27 (17.9)	41 (18.3)	4 (5.1)	9 (16.1)	
Did not know	27 (39.1)	60 (39.7)	118 (52.7)	59 (75.6)	38 (67.9)	
Did not answer	0 (0.0)	0 (0.0)	0 (0.0)	0 (0.0)	0 (0.0)	
	a	a/b	b/c	d	c/d	
Is there any restriction on intense physical activity for poliomyelitis patients?^*^						
Yes	30 (43.5)	72 (47.7)	83 (37.1)	16 (20.5)	15 (26.8)	< 0.001
No	17 (24.6)	22 (14.6)	39 (17.4)	4 (5.1)	4 (7.1)	
Did not know	22 (31.9)	57 (37.7)	102 (45.5)	58 (74.4)	37 (66.1)	
Did not answer	0 (0.0)	0 (0.0)	0 (0.0)	0 (0.0)	0 (0.0)	
	a	a/b	b	c	c	
Can people with PPS perform any type of physical activity?^*^						
Yes	48 (69.6)	104 (68.9)	95 (42.4)	23 (29.5)	19 (33.9)	< 0.001
No	1 (1.4)	2 (1.3)	12 (5.4)	1 (1.3)	1 (1.8)	
Did not know	20 (29)	45 (29.8)	117 (52.2)	54 (69.2)	36 (64.3)	
Did not answer	0 (0.0)	0 (0.0)	0 (0.0)	0 (0.0)	0 (0.0)	
	a	a	b/d	c	b/c	
Is there a need for clinical follow-up of patients, years after having been affected by poliomyelitis?^*^						
Yes	48 (69.6)	115 (76.2)	156 (69.6)	40 (51.3)	27 (48.2)	< 0.001
No	8 (11.6)	4 (2.6)	17 (7.6)	4 (5.1)	2 (3.6)	
Did not know	13 (18.8)	31 (20.5)	51 (22.8)	34 (43.6)	27 (48.2)	
Did not answer	0 (0.0)	1 (0.7)	0 (0.0)	0 (0.0)	0 (0.0)	
	a/b	a/c	b/c	d	d	
Is the most appropriate way to diagnose PPS based on symptomatology?^*^						
Yes	39 (56.5)	86 (57)	106 (47.3)	16 (20.5)	12 (21.4)	< 0.001
No	6 (8.7)	6 (4)	11 (4.9)	6 (7.7)	2 (3.6)	
Did not know	24 (34.8)	58 (38.4)	107 (47.8)	56 (71.8)	42 (75)	
Did not answer	0 (0.0)	1 (0.7)	0 (0.0)	0 (0.0)	0 (0.0)	
	a	a/b	b	c	c	
Because PPS is a poorly understood syndrome, there is still no effective form of treatment^*^						
Yes, agree	30 (43.5)	34 (22.5)	52 (23.2)	8 (10.3)	8 (14.3)	< 0.001
No, disagree	10 (14.5)	39 (25.8)	29 (12.9)	4 (5.1)	8 (14.3)	
Did not know	29 (42)	76 (50.3)	143 (63.8)	66 (84.6)	40 (71.4)	
Did not answer	0 (0.0)	2 (1.3)	0 (0.0)	0 (0.0)	0 (0.0)	
	a	a	b	c	b/c	
Is the poliovirus responsible for the onset of PPS?^*^						
Yes	13 (18.8)	42 (27.8)	76 (33.9)	11 (14.1)	8 (14.3)	< 0.001
No	31 (44.9)	35 (23.2)	32 (14.3)	2 (2.6)	4 (7.1)	
Did not know	25 (36.2)	73 (48.3)	115 (51.3)	65 (83.3)	44 (78.6)	
Did not answer	0 (0.0)	1 (0.7)	1 (0.4)	0 (0.0)	0 (0.0)	
		a	a	b	b	
Is PPS considered a neuromuscular disease?^*^						
Yes	44 (63.8)	104 (68.9)	138 (61.6)	28 (35.9)	19 (33.9)	< 0.001
No	1 (1.4)	6 (4)	4 (1.8)	1 (1.3)	0 (0.0)	
Did not know	24 (34.8)	40 (26.5)	82 (36.6)	49 (62.8)	37 (66.1)	
Did not answer	0 (0.0)	1 (0.7)	0 (0.0)	0 (0.0)	0 (0.0)	
	a/b	a	b	c	c	
Are the following main clinical manifestations presented by PPS patients: new weakness, fatigue and muscle and/or joint pain?^*^						
Yes	40 (58)	107 (70.9)	104 (46.4)	20 (25.6)	15 (26.8)	< 0.001
No	3 (4.3)	0 (0.0)	2 (0.9)	0 (0.0)	0 (0.0)	
Did not know	26 (37.7)	42 (27.8)	118 (52.7)	58 (74.4)	41 (73.2)	
Did not answer	0 (0.0)	2 (1.3)	0 (0.0)	0 (0.0)	0 (0.0)	
	a	a		b	b	
Can neuromuscular symptoms of PPS occur in limbs previously affected by poliomyelitis?^*^						
Yes	42 (60.9)	98 (64.9)	104 (46.4)	20 (25.6)	12 (21.4)	< 0.001
No	3 (4.3)	1 (0.7)	6 (2.7)	0 (0.0)	1 (1.8)	
Did not know	24 (34.8)	50 (33.1)	114 (50.9)	58 (74.4)	43 (76.8)	
Did not answer	0 (0.0)	2 (1.3)	0 (0.0)	0 (0.0)	0 (0.0)	
	a	a		b	b	
Can PPS be considered to be a progressive neuromuscular disease, presenting slow worsening of signs and symptoms?^*^						
Yes	35 (50.7)	62 (41.1)	98 (43.8)	20 (25.6)	15 (26.8)	< 0.001
No	7 (10.1)	26 (17.2)	14 (6.2)	1 (1.3)	2 (3.6)	
Did not know	27 (39.1)	60 (39.7)	112 (50)	56 (71.8)	39 (69.6)	
Did not answer	0 (0.0)	3 (2)	0 (0.0)	1 (1.3)	0 (0.0)	
	a/b	a	b	C	c	
Are you afraid to live with a person who has PPS?						
Yes	1 (1.4)	3 (2)	7 (3.1)	2 (2.6)	2 (3.6)	0.57
No	68 (98.6)	146 (96.7)	214 (95.5)	76 (97.4)	54 (96.4)	
Did not know	0 (0.0)	0 (0.0)	0 (0.0)	0 (0.0)	0 (0.0)	
Did not answer	0 (0.0)	2 (1.3)	3 (1.3)	0 (0.0)	0 (0.0)	
	a/b/c/d	a/e/f/g	b/e/h/i	c/f/h/j	d/g/i/j	
During your undergraduate course, did you have access to information on how to handle PPS?						
Yes	10 (14.5)	40 (26.5)	12 (5.4)	0 (0.0)	0 (0.0)	< 0.001
No	59 (85.5)	109 (72.2)	208 (92.9)	78 (100)	56 (100)	
Did not know	0 (0.0)	0 (0.0)	0 (0.0)	0 (0.0)	0 (0.0)	
Did not answer	0 (0.0)	2 (1.3)	4 (1.8)	0 (0.0)	0 (0.0)	
	a/b		b	c	c	
In your practice, have you ever provided a service for people with PPS?						
Yes	18 (26.1)	46 (30.5)	16 (7.1)	2 (2.6)	1 (1.8)	< 0.001
No	50 (72.5)	103 (68.2)	204 (91.1)	75 (96.2)	55 (98.2)	
Did not know	0 (0.0)	0 (0.0)	0 (0.0)	0 (0.0)	0 (0.0)	
Did not answer	1 (1.4)	2 (1.3)	4 (1.8)	1 (1.3)	0 (0.0)	
	a	a	b/d	b/c	c	

^*^ Question that composed the knowledge assessment test on paralytic poliomyelitis and PPS. Frequencies followed by the same letters, in the rows, did not differ.

With regard to the biological agent that causes poliomyelitis, some healthcare professionals reported that they did not know that the disease is caused by a virus (7.6%); and 4.0% of the participants stated that poliomyelitis is not caused by a virus. More than 90% of the physicians, physical therapists and nurses and approximately 70% of the nutritionists and psychologists correctly answered that poliomyelitis is caused by a virus. The chi-square test revealed that there was a significant association between healthcare professional category and knowledge of the biological agent that causes poliomyelitis (P < 0.001).

Out of the 578 participants approached, 78.3% of the physicians, 39.7% of the physical therapists, 54.5% of the nurses, 21.8% of the nutritionists and 25% of the psychologists knew that poliomyelitis can be spread through water and/or food contaminated with feces from a sick person. The chi-square test revealed that there was a significant association between healthcare professional category and knowing that poliomyelitis can be spread through water and/or food contaminated with feces from a sick person (P < 0.001).

Regarding the symptoms of poliomyelitis, 72.5% of the physicians, 41.1% of the physical therapists, 48.7% of the nurses, 26.9% of the nutritionists and 26.8% of the psychologists knew that poliomyelitis can cause gastrointestinal symptoms. The chi-square test revealed that there was a significant association between healthcare professional category and knowing that poliomyelitis can cause gastrointestinal symptoms (P < 0.001).

Regarding neuromuscular symptoms, more than 90% of the healthcare professionals knew that poliomyelitis can cause neuromuscular symptoms such as paralysis, paresis, muscle atrophy and weakness. The chi-square test did not reveal any significant association between healthcare professional category and knowing that poliomyelitis can cause neuromuscular symptoms (P = 0.262).

In relation to recovery of functional capacity after the acute poliomyelitis stage, 82.6% of the physicians, 70.2% of the physical therapists, 54% of the nurses, 33.3% of the nutritionists and 48.2% of the psychologists knew that after the acute poliomyelitis stage, patients can recover the functional capacity of affected structures. The chi-square test revealed that there was a significant association between healthcare professional category and knowing that patients can recover the functional capacity of affected structures (P < 0.001).

Regarding epidemiology, between 60 and 80% of the healthcare professionals knew that poliomyelitis is a disease that has not been eradicated worldwide. The chi-square test did not reveal any significant association between healthcare professional categories and knowing that poliomyelitis is a disease that has not been eradicated around the world (P = 0.406). Also, there was no significant association between health professional category with regard to knowing that a poliomyelitis vaccine is available (P = 0.133). Indeed, more than 90% of the healthcare professionals knew that a vaccine is available to prevent poliomyelitis.

With regard to treatment, 94.2% of the physicians, 78.8% of the physical therapists, 73.7% of the nurses, 33.3% of the nutritionists and 30.4% of the psychologists knew that poliomyelitis treatment can involve admission to an intensive care unit, due to respiratory impairment. The chi-square test revealed that there was a significant association between healthcare professional categories and knowing that poliomyelitis treatment can involve admission to an intensive care unit, due to respiratory impairment (P < 0.001).

Surprisingly, 15 healthcare professionals (2.6%) said that they were afraid to live with people with poliomyelitis (**Table 2**). The chi-square test revealed that there was a significant association between healthcare professional category and being afraid to live with people with poliomyelitis (P = 0.001).

Only 39.8% had received information about how to deal with patients with poliomyelitis during their undergraduate courses. Specifically, 59.5% of the physicians, 59.6% of the physical therapists, 40.6% of the nurses, 3.8% of the nutritionists and 8.9% of the psychologists had received information about poliomyelitis during their undergraduate courses. The chi-square test revealed that there was a significant association between healthcare professional category and provision of information about poliomyelitis during undergraduate courses (P < 0.001).

Regarding physical exercise, 92.8% of the physicians, 90.7% of the physical therapists, 79.0% of the nurses, 64.1% of the nutritionists and 66.1% of the psychologists responded that people with poliomyelitis sequelae can perform some kind of physical activity. The chi-square test revealed that there was a significant association between healthcare professional category and knowing about physical exercise (P < 0.001).

### Knowledge about postpoliomyelitis syndrome

The second part of the questionnaire was designed to assess knowledge about PPS. Out of the 578 participants approached, 243 (42%) had heard about PPS (**Table 3**). Specifically, 60.9% of the physicians, 67.5% of the physical therapists, 34.8% of the nurses, 12.8% of the nutritionists and 19.6% of the psychologists had heard about PPS. The chi-square test revealed that there was a significant association between healthcare professional category and having heard about PPS (P < 0.001).

Out of the 578 participants approached, 373 (65.6%) answered that they had received no information about PPS. While 53.6% of the physicians and 54.3% of the physical therapists had received information about PPS, only 26.3% of the nurses, 6.4% of the nutritionists and 14.3% of the psychologists had received information about PPS (**Table 3**). The chi-square test revealed that there was a significant association between healthcare professional category and having received information about PPS (P < 0.001).

Regarding pathophysiology, 302 (52.2%) did not know that the PPS affects only people who have had paralytic poliomyelitis in the past. While 33.3% of physicians and 42.4% of physical therapists knew that PPS is a disease that only affects patients who have had paralytic poliomyelitis, only 29% of the nurses, 19.2% of the nutritionists and 16.1% of the psychologists understood this. The chi-square test revealed that there was a significant association between healthcare professional category and knowing that PPS is a disease that only affects patients who have had paralytic poliomyelitis (P < 0.001).

In relation to restrictions that the patient must obey, 276 (47.8%) did not know whether there is any restriction on physical activity for people with poliomyelitis sequelae and only 216 (37.4%) knew that exercise should be limited among people with paralytic poliomyelitis sequelae (especially intense exercise). Specifically, 31.9% of the physicians, 37.7% of the physical therapists, 45.5% of the nurses, 74.4% of the nutritionists and 66.1% of the psychologists did not know about any restriction on intense physical activity for PPS patients. The chi-square test revealed that there was a significant association between healthcare professional category and knowing about any restriction on intense physical activity for PPS patients (P < 0.001).

Furthermore, 50% of the healthcare professionals answered that PPS patients can perform any type of physical activity and only 2.9% of them correctly answered that PPS patients cannot perform any type of physical activity (**Table 3**). The chi-square test revealed that there was a significant association between healthcare professional category and knowing that PPS patients cannot perform any type of physical activity (P < 0.001).

Regarding treatment, 386 (66.9%) believed that clinical follow-up is necessary, even years after acute poliomyelitis, while 156 (27.0%) did not know whether there is a need for clinical follow-up years after an individual has had polio. The chi-square test revealed that there was a significant association between healthcare professional category and knowing about the need for clinical follow-up of patients, years after having been affected by poliomyelitis (P < 0.001).

Also regarding treatment, approximately 61.1% of the healthcare professionals did not know whether there is an effective treatment for PPS. Specifically, 42.0% of the physicians, 50.3% of the physical therapists, 63.8% of the nurses, 84.6% of the nutritionists and 71.4% of the psychologists did not know about this issue. The chi-square test revealed that there was a significant association between healthcare professional category and this matter (P < 0.001).

With regard to diagnosis, 287 (49.7%) did not know that the most appropriate way to diagnose PPS is based on symptomatology. Specifically, 34.8% of the physicians, 38.4% of the physical therapists, 47.8% of the nurses, 71.8% of the nutritionists and 75% of the psychologists did not know about this issue. The chi-square test revealed that there was a significant association between healthcare professional category and this matter (P < 0.001).

Regarding PPS etiology, only 104 (18.0%) knew that poliovirus is not responsible for PPS. Specifically, 44.9% of the physicians, 23.2% of the physical therapists, 14.3% of the nurses, 2.6% of the nutritionists and 7.1% of the psychologists did not know about this issue. The chi-square test revealed that there was a significant association between healthcare professional category and this matter (P < 0.001).

Regarding PPS classification, 232 healthcare professionals (40.1%) did not know that PPS is a neuromuscular disease. Specifically, 34.8% of the physicians, 26.5% of the physical therapists, 36.6% of the nurses, 62.8% of the nutritionists and 66.1% of the psychologists did not know about this issue. The chi-square test revealed that there was a significant association between healthcare professional category and this matter (P < 0.001).

Regarding the main clinical manifestations presented by PPS patients, 285 of the healthcare professionals (49.3%) did not know that the main clinical manifestations of PPS are new weakness, fatigue and muscle and/or joint pain. Specifically, while 58% of the physicians, 70.9% of the physical therapists and 46.4% of the nurses knew about this topic, only 25.6% of the nutritionists and 26.8% of the psychologists knew that PPS patients can present with new weakness, fatigue and muscle and/or joint pain. The chi-square test revealed that there was a significant association between healthcare professional category and this matter (P < 0.001).

Furthermore, while 60.9% of the physicians, 64.9% of the physical therapists and 46.4% of the nurses knew that neuromuscular symptoms of PPS occur in limbs previously affected by poliomyelitis, only 25.6% of the nutritionists and 21.4% of the psychologists knew about this topic. The chi-square test revealed that there was a significant association between healthcare professional category and this matter (P < 0.001). A similar pattern was found for answers to question 27.

Surprisingly, 15 participants (2.6%) said that they were afraid to live with people with PPS. The chi-square test did not reveal any association between healthcare professional category and being afraid to live with a person who has PPS (P = 0.877).

Regarding the provision of information about this disease during the undergraduate course, only 62 participants (10.7%) reported that during their undergraduate courses they had had access to information about management of PPS, and only 83 healthcare professionals (14.4%) reported that they had already provided services to people with PPS (**Table 3**). The chi-square test did not reveal any association between healthcare professional category and these topics (P < 0.001).

### Knowledge of the professionals about paralytic poliomyelitis and PPS

**Figure 1:**
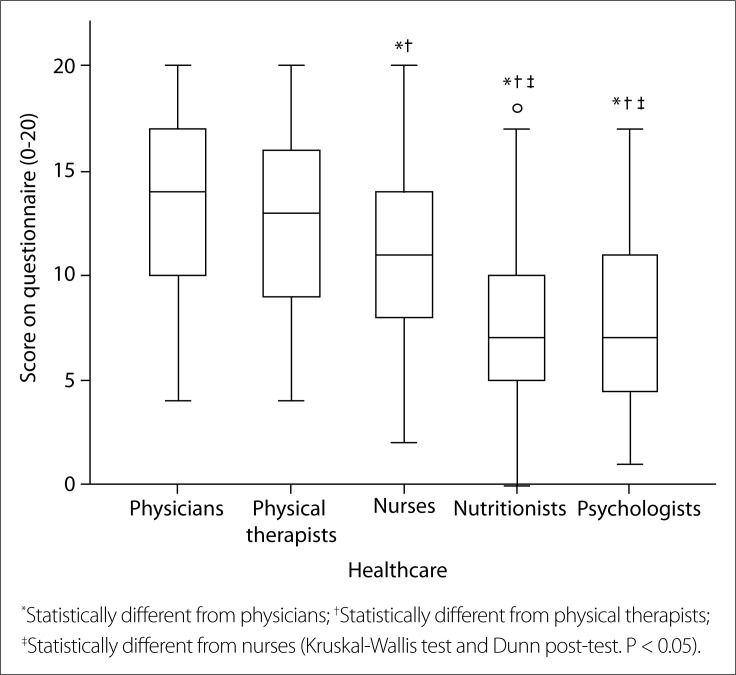
Questionnaire result (0-20) separated according to healthcare professional categories.

With regard to the questionnaire that was created, the professionals scored on average 11.0 ± 4.4 (which corresponded to approximately 55% of the total score), out of a maximum score of 20. Specifically, the Kruskal-Wallis test [X^2^(4) = 107.500; P < 0.001] demonstrated that the knowledge of the physicians, physical therapists and nurses was significantly higher than that of the nutritionists and psychologists (P < 0.05, **Figure 1**); and that the knowledge of the physicians and physical therapists was significantly higher than that of the nurses, nutritionists and psychologists (P < 0.05).

**Figure 2:**
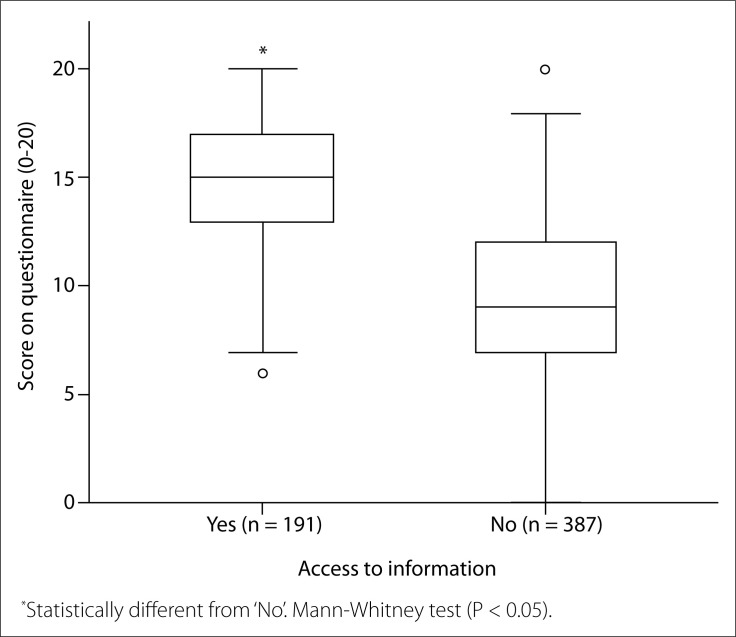
Questionnaire result (0-20) separated according to healthcare professionals with (Yes; n = 191) or without (No; n = 387) access to information about poliomyelitis and post-poliomyelitis syndrome.

We also found that healthcare professionals who had received previous information about poliomyelitis and PPS had significantly higher scores than those who had never received information (P < 0.001). On average, this difference was approximately 28.6% (**Figure 2**). Five volunteers did not respond to this question.

## DISCUSSION

This study evaluated knowledge about paralytic poliomyelitis and PPS among healthcare professionals (physicians, physical therapists, nurses, nutritionists and psychologists). Considering that success in treating a disease depends on the knowledge level that healthcare professionals have, with regard to etiology, signs and symptoms and management of the disease, studies with the aim of investigating the knowledge of these professionals about a particular disease are important. Despite finding misconceptions about poliomyelitis and PPS in all the healthcare professional categories assessed, our results revealed that physicians, physical therapists and nurses present higher knowledge about poliomyelitis and PPS than nutritionists and psychologists. Furthermore, we found that those who had received previous information about poliomyelitis and PPS had significantly higher scores than those who had never received information.

Previous studies have investigated healthcare professionals’ knowledge about certain diseases. Morin et al.^33^ investigated physicians’ knowledge about cryptosporidiosis and reported that these professionals did not have adequate knowledge about this disease. Vancini et al.^26^ investigated the knowledge of physical education professionals about epilepsy and also found low knowledge among these professionals. Regarding paralytic poliomyelitis and PPS, de Lira et al.^8^ found that physical education professionals had low knowledge about these diseases. Therefore, our results are in line with the literature.

The low level of knowledge about paralytic poliomyelitis and PPS can be explained because poliomyelitis is a disease that has been eradicated in various countries (including Brazil).^34^ In addition, PPS is a relatively unknown disease and physicians commonly confound the signs and symptoms of this disease with the aging process. Only recently, as a result of an initiative led by professionals at the Federal University of São Paulo, PPS was included in the International Classification of Diseases.^35^ Therefore, our findings were expected.

It is necessary to highlight that the low levels of knowledge demonstrated by these healthcare professionals (especially by the nutritionists and psychologists) are worrying, because it is reasonable to assume that patient care procedures can be influenced by the professional knowledge level. In this context, we found that healthcare professionals who had received previous information about poliomyelitis and PPS had significantly higher scores than those who had never received information. This result suggests that universities should include information about poliomyelitis and PPS in their undergraduate curricula, in order to improve the students’ knowledge about poliomyelitis and, consequently, as future professionals. Not least, this result suggests that continuing education programs should be implemented as a government initiative. Furthermore, the low knowledge about paralytic poliomyelitis and PPS is worrying, because it is reasonable to assume that low knowledge could decrease professionals’ ability to provide counseling about the importance of vaccines.

We also found that physicians, physical therapists and nurses presented higher knowledge than psychologists and nutritionists, as demonstrated by the scores in the knowledge assessment test. Considering that approximately 90% of the physicians, physical therapists and nurses had access to information on how to deal with poliomyelitis in their undergraduate studies and that this proportion fell to approximately 50% among the nutritionists and psychologists, this result was expected. Indeed, the fact that psychologists and nutritionists demonstrate less knowledge about the disease can be explained by the lack of material dedicated to infectious diseases in the undergraduate curricula of such courses. In particular, it is extremely important that physicians have high knowledge about the criteria for diagnosing PPS, because it is a syndrome for which the symptoms include new muscle weakness and muscle fatigue, among patients who have a history of paralytic poliomyelitis.^22^,^25^ However only 33% of the physicians knew that PPS affects individuals who have had polio in the past, and this is important because it is the main diagnostic criterion.

With regard to knowing that PPS is considered to be a progressive neuromuscular disease, with slow worsening of signs and symptoms, approximately 51% of the physicians had correct knowledge, while 42% of the physical therapists, 43% of the nurses, 26% of the nutritionists and 27% of the psychologists had correct knowledge. This shows that there are wide differences in knowledge among healthcare professionals. This result can probably be explained by the fact that physicians, physical therapists and nurses are directly involved in diagnosis and treatment in hospitals and clinics^25^ and, as mentioned above, by the presence of material dedicated to infectious diseases in the undergraduate curricula. On the other hand, the participation of nutritionists and psychologists is associated with ameliorating secondary symptoms, such as recent body mass gain^36^ and mood disorders.^34^

Although paralytic poliomyelitis has been eradicated in most countries (including Brazil), there are still some countries with new cases of paralytic poliomyelitis.^37^ In 2018, the World Health Organization (WHO) recorded 32 cases of poliomyelitis derived from wild poliovirus and 105 cases of poliomyelitis derived from circulating vaccine-derived poliovirus.^37^ Furthermore, in the recent humanitarian crisis due to the civil war in Syria, the WHO officially acknowledged an outbreak on October 29, 2013. In May 2014, the WHO declared polio to be a global health emergency for the first time in the organization’s history. This was a substantial challenge to the 25-year-old efforts of the Global Polio Eradication Initiative, which had been successful in eliminating polio from Syria in 1995.^38^ Specifically in Brazil, the authorities reported difficulties in attaining vaccine coverage in the 2018 campaign against paralytic poliomyelitis. ^39^ For this reason, the Brazilian authorities have launched another vaccine campaign in order to reach the vaccine coverage recommended by the WHO.^39^ Altogether, this information highlights the need to improve knowledge about poliomyelitis and PPS among healthcare professionals.

Our study had some limitations. Firstly, like all studies in which questionnaires are used, the present results rely on the honesty and level of recall of the respondents. Secondly, the reliability and validity of the instrument used to gather the data for this study has not been determined, although the questionnaire was previously evaluated by two experienced researchers. Nevertheless, we believe that these limitations do not prevent us from drawing conclusions from this study.

## CONCLUSION

Our study showed that, overall, there is a lack of knowledge about PPS and poliomyelitis, especially among psychologists and nutritionists. Therefore, the services provided by these professionals may become compromised. Furthermore, government initiatives should be implemented to increase knowledge among healthcare professionals.
